# Disease prevalence among young dogs in Grand Tunis, Tunisia: A retrospective study

**DOI:** 10.14202/vetworld.2019.489-495

**Published:** 2019-04-02

**Authors:** Ghada Tagorti

**Affiliations:** Department of Small Animal Medicine and Surgery, National School of Veterinary Medicine, Manouba University, 2020 Sidi Thabet, Tunisia

**Keywords:** disease, dog, epidemiology, juvenile, prevalence, Tunisia

## Abstract

**Aim:**

A retrospective study was undertaken to determine the occurrence, and the distribution of the most common clinical conditions of young dogs encountered at the National School of Veterinary Medicine clinic, Tunisia, from September 2012 to July 2013, based on sex, age, breeds, and season variation.

**Materials and Methods:**

A total of 515 cases were examined, and 11 clinical conditions were recorded. Clinical examination was performed. X-ray examination and necropsy were carried out only when needed.

**Results:**

Of the 515, 298 cases (57.86%) were male, while 217 (42.14%) were female. The breed-wise difference in the occurrence of various health problems was statistically significant. Nevertheless, no significant association was found between the occurrence of a disease and age. The commonly found clinical conditions were traumatic injuries (22.72%), ectoparasitic infections (20.58%), and gastroenteritis (13.40%). The occurrence of diseases was the highest (60.19%) in the wet season (September-February) followed by 39.81% in the dry season (March-July).

**Conclusion:**

The current study presents the first recorded data about the major clinical conditions of young dogs in Tunisia. These findings can be used to develop more effective disease management and control strategies.

## Introduction

Domestic dog is the most widespread, abundant, and sociable canids. Since 15,000 years ago, the relationship between dogs and humans has been mutually beneficial [1]. Domestic dogs were kept as social companions and/or working services (e.g., hunting dogs, guardian dogs, therapy dogs, and police dogs) [2]. Currently, the dog breeding increased, to reach in 2016 more than 671 thousand dogs in Tunisia [3]. Thus, dogs often go beyond the boundaries of the house to develop animal instincts and prevent undesirable behavior correlated to stress and poor welfare [4]. As a result, environmental conditions will affect the ability of dogs to cope with change. Therefore, dogs will face health issue and disease management, especially during the juvenile stage while the immune system is immature and of weak efficacy [5]. In addition, the heterogeneity of purebreds creates a great diversity on the function of the immune system between various canine breeds [6]. With such background, the interaction of young dogs toward pathogen agent and the environment is specific to each, and these components can shift the balance toward or away from emerging diseases.

On the other hand, the Grand Tunis, which is defined geographically as the area including four governorates (Ben Arous, Tunis, Ariana, and Manouba) located in the North of Tunisia, is characteristically semi-arid with a mild winter. These climatic conditions are generally conducive for infectious diseases. Consequently, there has been a good deal of research conducted on companion animal diseases. However, the majority had focused on the surveillance and control of diseases in adult dogs.

Hence, to reduce the gap in our knowledge of the distribution of clinically important diseases of young dogs, this retrospective study was carried out to evaluate the major disease conditions diagnosed at the National School of Veterinary Medicine clinic. This study is the first to report on disease profiles of juvenile dogs in Tunisia.

## Materials and Methods

The retrospective data were retrieved from the clinical case records of owned dogs of the National School of Veterinary Medicine clinic, located in the Ariana Governorate in Tunisia (longitude 10°2’52.587” E and latitude 36°54’26.219” N) from September 2012 to July 2013. A total number of 515 young dogs aged under 7 months (juvenile phase) were recorded from 2291 dogs overall after the exclusion of cases for routine deworming and vaccinations. The distribution patterns of cases were according to sex, age, breeds, and season (August is not included due to summer break) to represent the Grand Tunis area. Diagnosis was based on history, physical examination, clinical signs, and sometimes postmortem findings. Detailed clinical examination was performed using visual examination, rectal temperature, pulse, respiration rates, and examination of different organs and systems using palpation, percussion, and auscultation when needed [7]. Extension and flexion, needle puncture, ophthalmic, and X-ray examination were also performed when required. For diagnostic purposes, fecal samples, skin scrapings, blood, and urine samples were examined. Necropsy was carried out on dead young dogs to record the gross lesions.

### Statistical analysis

Data were summarized, reported as percentages, and subjected to Fisher’s exact test. All analyses were performed with analytical software (SPSS version 20.0, SPSS Inc., Chicago, IL, USA). Values of p≤0.05 were considered as statistically significant.

## Results and Discussion

**Figure-1:**
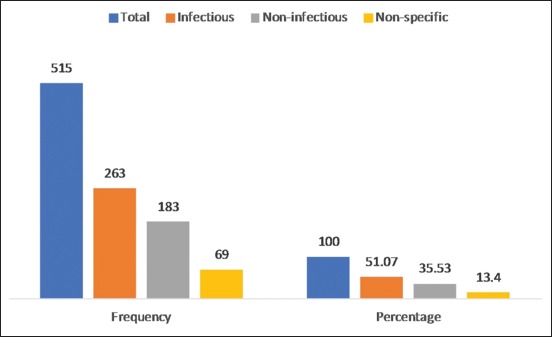
Classification of clinical conditions of young dogs.

**Figure-2:**
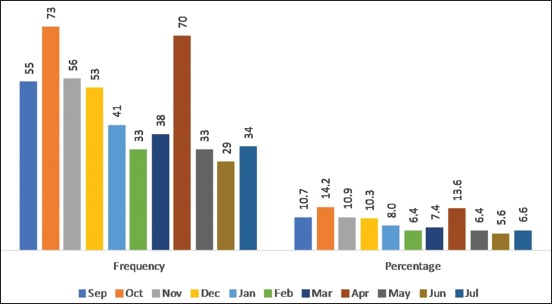
Monthly distribution of clinical cases of young dogs.

The present study had given an overall idea about the prevalence of the most commonly occurring juvenile canine diseases presented at the National School of Veterinary Medicine clinic in Tunisia during the period going from September 2012 to July 2013. The juvenile problems had an overall prevalence of 22.5% during this period. Similar findings were reported in a study in Bangladesh, and the recorded prevalence was 36.1% [8]. The highest incidence of juvenile affections was recorded in the males (57.86%), with 42.14% of cases for females (p=0.012). This finding may coincide with the pet owner preference and gender stereotypes. It was hypothesized that men with their masculinity and independence characteristics predicted a preference for male dogs [9]. Of 515 young dogs presented with different clinical conditions, 286 (55.53%) were aged between 1 and 3 months as compared to the group aged more than 3 months (44.47%). However, the analysis showed no statistically significant association between age group and clinical conditions. To depict the morbidity profile by breeds of young dogs, a total of eight breeds were recorded as the most common in the studied area. Therefore, breed-specific proportional morbidity rates revealed that American Staffordshire Terrier (27.18%), German Shepherd (18.25%), mixed breed (14.56%), Rottweiler (12.43%), Poodle (5.83%), Spaniel (6.02%), and Labrador (4.47%) were affected (Table-1). This observed incidence could be related to the population distribution based on the upsurge in the acquisition of these specific breeds for security or breeding purposes. Besides, selective breeding and the repeated use of popular sires could have increased the susceptibility of developing diseases in domestic dogs [10]. In the current study, of 515 young dogs, 11 clinical conditions were diagnosed, representing infectious diseases (51.07%) followed by non-infectious (35.53%) and non-specific diseases or diseases with multiples causes (13.4%) (Figure-1). Consequently, infectious diseases were the major causes of young dogs’ diseases with predominantly ectoparasitic (20.58%) and endoparasitic infections (11.65%). In addition, traumatic injuries (22.72%) and nutritional secondary hyperparathyroidism (NSH) (7.77%) were the most common non-infectious diseases reported. Gastroenteritis represented the non-specific group with 13.40% of clinical cases. Nevertheless, lower prevalence of infectious and non-infectious diseases was recorded by earlier studies [8,11]. This variation may be due to different geographical regions and periods. In addition, diseases were prominent in the wet season (60.19%) due to the multiplication of etiologic agents in these environmental conditions (Figure-2).

**Table-1:** Breed distribution of clinical conditions of young dogs.

Clinical condition	Total n (%)	Breeds								p-value

		American Staffordshire terrier	G.S	Spaniel	Poodle	Mixed	Rottweiler	Labrador	Others	
Infectious										
Parvovirosis	54 (10.49)	13	33	2	0	0	6	0	0	0.000*
Ehrlichiosis	11 (2.14)	9	0	0	0	0	2	0	0	0.005*
RT infections	5 (0.97)	0	0	0	0	0	1	3	1	0.907
Eye disorders	27 (5.24)	4	2	2	3	2	4	4	6	0.975
Endoparasitic I	60 (11.65)	20	6	6	0	12	12	0	4	0.000*
Ectoparasitic I	106 (20.58)	30	13	5	6	22	13	5	12	0.009*
Subtotal	263 (51.07)	76	54	15	9	36	38	12	23	
Non-infectious										
Hip dysplasia	11 (2.14)	1	8	0	0	0	2	0	0	0.027*
Traumatic injuries	117 (22.72)	29	12	7	13	26	12	3	15	0.000*
NSH	40 (7.77)	16	10	4	0	3	4	0	3	0.006*
Congenital anomalies	15 (2.91)	5	0	1	0	3	1	2	3	0.826
Subtotal	183 (35.54)	51	30	12	13	32	19	5	21	
Non-specific										
Gastroenteritis	69 (13.40)	13	10	4	8	7	7	6	14	0.687
Subtotal	69 (13.40)	13	10	4	8	7	7	6	14	0.687
Grand total	515 (100.00)	140	94	31	30	75	64	23	58	0.000*

G.S = German Shepherd, RT = Respiratory tract, NSH = Nutritional secondary hyperparathyroidism, I = Infections. Asterisks (*) indicate a significant association

### Infectious diseases

#### Parvovirosis

Canine parvovirus, contagious acute enteritis, occurred more commonly in dogs younger than 3 months of age in the current study which might be due to insufficient immunity (Table-2) [12]. On the other hand, a highly significant (p=0.000) association was observed between the breeds and occurrence of parvovirus.

**Table-2:** Age distribution of clinical conditions of young dogs.

Clinical condition	Total n (%)	Age		p-value

		1-3 months n (%)	>3 months n (%)	
Infectious				
Parvovirosis	54 (10.49)	38 (70.37)	16 (29.63)	0.049*
Ehrlichiosis	11 (2.14)	3 (27.27)	8 (72.73)	0.400
RT infections	5 (0.97)	4 (80.00)	1 (20.00)	0.545
Eye disorders	27 (5.24)	9 (33.33)	18 (66.67)	0.277
Endoparasitic I	60 (11.65)	60 (100.00)	0 (0.00)	0.000*
Ectoparasitic I	106 (20.58)	50 (47.17)	56 (52.83)	0.784
Subtotal	263 (51.07)	164 (62.36)	99 (37.64)	
Non-infectious				
Hip dysplasia	11 (2.14)	1 (9.09)	10 (90.91)	0.069
Traumatic injuries	117 (22.72)	47 (40.17)	70 (59.83)	0.150
NSH	40 (7.77)	33 (82.50)	7 (17.50)	0.004*
Congenital anomalies	15 (2.91)	8 (53.33)	7 (46.67)	1.000
Subtotal	183 (35.54)	89 (48.63)	94 (51.37)	
Non-specific				
Gastroenteritis	69 (13.40)	33 (47.83)	36 (52.17)	0.866
Subtotal	69 (13.40)	33 (47.83)	36 (52.17)	0.866
Grand total	515 (100.00)	286 (55.53)	229 (44.47)	0.081

RT = Respiratory tract, NSH = Nutritional secondary hyperparathyroidism, I = Infections. Asterisks (*) indicate a significant association

#### Ehrlichiosis

Breed-wise incidence of ehrlichiosis was recorded higher in American Staffordshire Terrier. This observation was in agreement with the findings of Lakshmanan [13] who reported a higher prevalence of ehrlichiosis in purebred dogs. Therefore, this discrepancy in the breed predisposition might be due to immunological competence and susceptibility of different breeds to tick infestation [14]. Even that all breeds are prone to ehrlichiosis, some breeds such as German Shepherd dogs are more predisposed due to the inherent breed inability of blast formation [15]. Moreover, results suggested that there was no association between the occurrences of ehrlichiosis with sex, age, or season.

#### Respiratory tract (RT) infections

The RT is constantly exposed to infectious agents that can reach the upper and lower RT. Disease prevalence of the respiratory system in the present study was 0.97% close to that reported in a previous study [16]. The data analyzed found no association between the infection and different parameters.

#### Eye disorders

The reported prevalence of eye problems, in this study, was 5.24% in young dogs that agreed with the results of Sarker *et al*. [16]. It affects males as well as females, regardless of age and season. It is rare that gender factor involves in the incidence of these kinds of pathologies and a few ocular diseases are specific to young dogs; still, it may occur at any age, except for glaucoma, which is almost reserved for older animals [17].

#### Endoparasitic infections

The prevalence of endoparasites found in this study was 11.65%. According to other studies, the estimated worldwide prevalence of endoparasitic infections varies from 5% to 70% [18]. Therefore, for young dogs, the prevalence of 3.84% and 20% were recorded in Bangladesh and Nigeria, respectively [11,18]. This difference depends on many factors such as the geographical location, sampling protocols, and anthelmintic usage. Moreover, this study demonstrated that young dogs aged between 1 and 3 months are more likely to be infected with helminths and protozoa than older dogs (p = 0.000). It conforms to the earlier studies where infections with *Toxocara canis*, *Isospora* spp., and *Giardia* spp. represent the highest rates between the 6- and 12-week-old pups which are partially due to transmammary and lactogenic transmission [19]. On the basis of breed, the result showed that the American Staffordshire Terrier had higher prevalence rates of endoparasites (33.33%) than the mixed breeds (20%) and Rottweiler (20%). Analysis showed a strong association (p=0.000) between breeds and the prevalence of endoparasites. American Staffordshire Terrier and Rottweiler may be less immune to the parasites than the mixed breeds.

On the other hand, mixed breeds are usually more free to roam from one place to another, and therefore get exposed [20]. Nevertheless, no significant breed difference was found in another study [21].

#### Ectoparasitic infections

Ectoparasitic infections were recorded on 57 male dogs (53.77%) and 49 female dogs (46.23%). Nevertheless, no statistically significant difference was observed between host gender and ectoparasite infestation (Table-3). Similar findings were reported in other studies [22,23]. Although ectoparasites were noted in young dogs throughout all the period of the study, they were more prevalent in the wet season, with a significant association between season and infestation (Table-4). This seasonal influence was observed in other countries as well [24]. It could be associated with variations in temperature and humidity required for ectoparasite survival and reproduction, which may vary between geographical regions. On the other hand, dogs kept indoors are less exposed to ectoparasites compared to hunting dogs [25]. However, these two categories were equally affected in the current study. Furthermore, compared to purebred dogs, mixed breed dogs were less infested (79.25% vs. 20.75%).

**Table-3:** Sex distribution of clinical conditions of young dogs.

Clinical condition	Total n (%)	Sex		p-value

		Female n (%)	Male n (%)	
Infectious				
Parvovirosis	54 (10.49)	22 (40.74)	32 (59.26)	0.440
Ehrlichiosis	11 (2.14)	6 (54.55)	5 (45.45)	1.000
RT infections	5 (0.97)	2 (40.00)	3 (60.00)	1.000
Eye disorders	27 (5.24)	13 (48.15)	14 (51.85)	1.000
Endoparasitic I	60 (11.65)	25 (41.67)	35 (58.33)	0.464
Ectoparasitic I	106 (20.58)	49 (46.23)	57 (53.77)	0.680
Subtotal	263 (51.07)	117 (44.49)	146 (55.51)	
Non-infectious				
Hip dysplasia	11 (2.14)	0 (0.00)	11 (100.00)	0.014*
Traumatic injuries	117 (22.72)	50 (42.74)	67 (57.26)	0.296
NSH	40 (7.77)	16 (40.00)	24 (60.00)	0.500
Congenital anomalies	15 (2.91)	3 (20.00)	12 (80.00)	0.135
Subtotal	183 (35.54)	69 (37.70)	114 (62.30)	
Non-specific				
Gastroenteritis	69 (13.40)	31 (44.93)	38 (55.07)	0.612
Subtotal	69 (13.40)	31 (44.93)	38 (55.07)	0.612
Grand total	515 (100.00)	217 (42.14)	298 (57.86)	0.012*

RT = Respiratory tract, NSH = Nutritional secondary hyperparathyroidism, I = Infections. Asterisks (*) indicate a significant association

**Table-4:** Season distribution of clinical conditions of young dogs.

Clinical condition	Total n (%)	Season		p-value

		Wet (September-February) n (%)	Dry (March-July) n (%)	
Infectious				
Parvovirosis	54 (10.49)	33 (61.11)	21 (38.89)	0.333
Ehrlichiosis	11 (2.14)	8 (72.73)	3 (27.27)	0.400
RT infections	5 (0.97)	4 (80.00)	1 (20.00)	0.545
Eye disorders	27 (5.24)	15 (55.56)	12 (44.44)	0.789
Endoparasitic I	60 (11.65)	39 (65.00)	21 (35.00)	0.139
Ectoparasitic I	106 (20.58)	72 (67.92)	34 (32.08)	0.012*
Subtotal	263 (51.07)	171 (65.02)	92 (34.98)	
Non-infectious				
Hip dysplasia	11 (2.14)	9 (81.82)	2 (18.18)	0.193
Traumatic injuries	117 (22.72)	64 (54.70)	53 (45.30)	0.515
NSH	40 (7.77)	19 (47.50)	21 (52.50)	1.000
Congenital anomalies	15 (2.91)	8 (53.33)	7 (46.67)	1.000
Subtotal	183 (35.54)	100 (54.64)	83 (45.36)	
Non-specific				
Gastroenteritis	69 (13.40)	39 (56.52)	30 (43.48)	0.498
Subtotal	69 (13.40)	39 (56.52)	30 (43.48)	0.498
Grand total	515 (100.00)	310 (60.19)	205 (39.81)	0.001*

RT = Respiratory tract, NSH = Nutritional secondary hyperparathyroidism, I = Infections. Asterisks (*) indicate a significant association

### Non-infectious diseases

#### Hip dysplasia

Hip dysplasia, a common developmental disorder, had affected 11 male dogs (100%) in the present study, compared to other studies where only 59.55% of male dogs were affected [26]. This could be related to over breeding, which leads to the occurrence of hip dysplasia or to the fact that male neutered dogs may be at increased risk for the development of hip dysplasia than females, especially of neutering at an age younger than 6 months [26,27]. Still, multiple prevalence studies showed no sex predilection [28]. Besides, clinical signs are usually evident at 4-12-month-old dogs [29]; this explains the high incidence in the group of age older than 3 months (90.91%). Nevertheless, no significant association was reported. Furthermore, any size or breed of dog can be affected; however, the condition is commonly evaluated in purebred and large breed to check the possibility of breeding. This explains the finding where German Shepherd, Rottweiler, and American Staffordshire Terrier represented 72.73%, 18.18%, and 9.09%, respectively (p=0.027). On the other hand, season had a non-significant effect on the distribution of such condition.

#### Traumatic injuries

Young dogs are faced with dangers and traumatic injuries from a minor wound to fracture. However, a breed distribution was observed (Table-1). This is in part due to population distribution to the owner’s preference. Nevertheless, no association between clinical cases and other parameters has been established.

#### NSH

This metabolic condition is caused by a high dietary intake of phosphorus and chronic decrease in calcium. Puppies and large breeds were found to be, especially, susceptible to it due to the high calcium requirement [30,31]. This observation agrees with the current study where the highest occurrence was recorded in American Staffordshire Terrier.

#### Congenital anomalies

Usually, congenital anomalies tend to manifest after some time and so quite frequently a delay in consulting to the veterinarian since no subtle signs are there. Interestingly, in the current study, some cases of congenital anomalies were reported in 2.91% of cases.

### Non-specific diseases

#### Gastroenteritis

The present work showed that gastroenteritis had a prevalence of 13.40% of the total diseased young dogs. Contrarily, other findings had reported a prevalence of 52% in Turkey and 56.5% in Egypt [32,33]. This could be explained to a difference in geographical distribution. Besides, no association between the age and frequency of gastrointestinal disorders was reported in the current study. This result is in accordance with Hubbard *et al*.[34].

## Conclusion

The present study was the first recorded data about the prevalence of clinical conditions in young dogs admitted to the National School of Veterinary Medicine clinic, Tunisia, during the period from September 2012 to July 2013. It was concluded that traumatic injuries, ectoparasitic infections, and gastroenteritis were the major health issue among young dogs. Sex, breeds, and season had a significant effect on the distribution of juvenile canine diseases. Therefore, medical awareness of the owners should be increased to improve the ectoparasites control measures, deworming procedure, and vaccination status as well as to dedicate adequate attention to young dogs.

## Author’s Contributions

GT was responsible for all parts of this study. The author read, finalized, and approved the manuscript.
